# The Madrid Posterior Component Separation: An Anatomical Approach for Effective Reconstruction of Complex Midline Hernias

**DOI:** 10.3389/jaws.2024.12928

**Published:** 2024-06-10

**Authors:** Marcello De Luca, Manuel Medina Pedrique, Sara Morejon Ruiz, Joaquin M. Munoz-Rodriguez, Alvaro Robin Valle de Lersundi, Javier Lopez-Monclus, Luis Alberto Blázquez Hernando, Miguel Angel Garcia-Urena

**Affiliations:** ^1^ UOC Chirurgia Generale Oncologica e Mininvasiva, Azienda Ospedaliera Universitaria, University of Naples Federico II, Naples, Campania, Italy; ^2^ Servicio de Cirugía General y Aparato Digestivo, Hospital Universitario del Henares, Fundación Investigación e Innovación Biomédica H. Santa Sofía- H del Henares, Madrid, Spain; ^3^ Grupo de Investigación de Pared Abdominal Compleja, Universidad Francisco de Vitoria, Madrid, Spain; ^4^ Servicio de Cirugía General y Aparato Digestivo, Hospital Universitario Puerta de HIerro, Madrid, Spain; ^5^ Servicio de Cirugía General y Aparato Digestivo, Hospital Universitario Ramón y Cajal, Madrid, Spain

**Keywords:** Madrid APPROACH, Madrid posterior component separation, Madrid TAR, posterior component separation, posterior rectus sheath release

## Abstract

**Introduction:**

In recent years, Posterior Component Separation (PCS) with the Madrid modification (Madrid PCS) has emerged as a surgical technique. This modification is believed to enhance the dissection of anatomical structures, offering several advantages. The study aims to present a detailed description of this surgical technique and to analyse the outcomes in a large cohort of patients.

**Materials and Methods:**

This study included all patients who underwent the repair of midline incisional hernias, with or without other abdominal wall defects. Data from patients at three different centres specialising in abdominal wall reconstruction was analysed. All patients underwent the Madrid PCS, and several variables, such as demographics, perioperative details, postoperative complications, and recurrences, were assessed.

**Results:**

Between January 2015 and June 2023, a total of 223 patients underwent the Madrid PCS. The mean age was 63.4 years, with a mean BMI of 33.3 kg/m^2^ (range 23–40). According to the EHS classification, 139 patients had a midline incisional hernia, and 84 had a midline incisional hernia with a concomitant lateral incisional hernia. According to the Ventral Hernia Working Group (VHWG) classification, 177 (79.4%) patients had grade 2 and 3 hernias. In total, 201 patients (90.1%) were ASA II and III. The Carolinas Equation for Determining Associated Risks (CeDAR) was calculated preoperatively, resulting in 150 (67.3%) patients with a score between 30% and 60%. A total of 105 patients (48.4%) had previously undergone abdominal wall repair surgery. There were 93 (41.7%) surgical site occurrences (SSO), 36 (16.1%) surgical site infections (SSI), including 23 (10.3%) superficial and 7 (3.1%) deep infections, and 6 (2.7%) organ/space infections. Four (1.9%) recurrences were assessed by CT scan with an average follow-up of 23.9 months (range 6–74).

**Conclusion:**

The Madrid PCS appears to be safe and effective, yielding excellent long-term results despite the complexity of abdominal wall defects. A profound understanding of the anatomy is crucial for optimal outcomes. The Madrid modification contributes to facilitating a complete retromuscular preperitoneal repair without incision of the transversus abdominis. The extensive abdominal wall retromuscular dissection obtained enables the placement of very large meshes with minimal fixation.

## Introduction

All patients undergoing abdominal surgery with a laparotomic incision are exposed to the risk of developing an incisional hernia (IH) [1–3]. The treatment of large IH, especially in complex abdominal cases, has been and continues to be a significant challenge for surgeons [4]. Over the past decades, various techniques have been described based on the prosthetic material reinforcement and the anatomical plane used, each attempting to provide advantages over previous techniques.

The use of the retro-muscular and preperitoneal planes, described by Rives and Stoppa, allows for the reconstruction of the abdominal wall using a non-absorbable prosthetic material, positioning it without direct contact with the intestinal loops and avoiding subcutaneous dissection [5,6]. However, this technique cannot be used for larger midline defects that require dissection beyond the *linea semilunaris* or for lateral IH.

To overcome this limitation, Carbonell devised the posterior component separation (PCS) in 2008, and Novitsky modified it in 2012 by introducing the Transversus Abdominis Release (TAR) [7,8]. While both, anterior and posterior component separation are based on the release of one of the lateral abdominal wall muscles, Heniford proposed to enter the preperitoneal space without adding any muscular release [9,10]. The goal of all these techniques remained essentially the same. The researchers aimed to obtain an extensive dissection in the retro-muscular and preperitoneal planes allowing the placement of a large mesh as a closure reinforcement.

Subsequently, after mastering the technique, improving the knowledge of prosthetic materials, and conducting anatomical studies on cadavers, we suggested some modifications to the original TAR [11,12]. The combination of permanent and absorbable prosthetic materials has been defined as the “Madrid APPROACH” (Absorbable Posterior Reinforcement of Permanent mesh Of A Complex Hernia) [13], and the preservation of the transversus abdominis (TA) muscle fibres through the release of the posterior rectus sheath (PRS), named the “Madrid modification” [14], has been introduced as an effective and safe technique [11].

The aim of this multicentre study is to provide a detailed description of the Madrid posterior component separation (Madrid PCS), including an analysis of the results from a large cohort of patients to update previously published results.

## Materials and Methods

From January 2015 to June 2023, all coecutive patients undergoing abdominal wall surgery for midline incisional hernias were enrolled. The inclusion criterion was the use of the Madrid PCS technique; any other form of abdominal wall reconstruction for midline IH was excluded. This study involved three specialised abdominal wall surgery centres located in Madrid. Patients were prospectively entered into a shared database on Redcap.

Demographic variables were collected for all patients, including age, sex, BMI, comorbidities, CeDAR (Carolinas Equation for Determining Associated Risk), ASA score, type of previous surgery and the number of previous attempts at abdominal wall reconstruction. The characteristics of midline IH were recorded according to the EHS classification (EHS M1-M5) [15], focusing on size and location. Additionally, all midline IH associated with lateral defects (EHS L1-L4) or with inguinal hernia were also recorded. Finally, variables related to bridging and reinsertion of the TA muscle were documented.

Postoperative variables, including systemic or local surgical complications (SSO, SSI, and SSOPI), were classified according to the Clavien-Dindo Classification (CDC) [16]. Intensive care unit stay, length of hospitalisation and readmission were also analysed. Clinical follow-up was conducted at 6 weeks, 3 months, 6 months, and then annually. A CT scan was performed when clinical examination raised doubt about a recurrence. Late complications, such as chronic seroma, chronic prosthesis infection, chronic pain, bulging, recurrence, intestinal obstruction, and mortality, were recorded.

This study was reported in line with the STROBE statement [17]. The study was approved by the Research Ethics Committee of Francisco de Vitoria University (39/2019) and the Institutional Review Board (37/2022). The patients provided written informed consent to participate in this study.

### Surgical Technique

All patients followed a standardised preoperative optimisation programme that comprised endocrinological and nutritional assessments, respiratory physiotherapy, and abstinence from smoking for a minimum of 1 month prior to surgery. While weight loss was strongly recommended, it was not mandatory. Since 2018, preoperative botulinum toxin has been regularly administered for defects greater than 9 cm, and pneumoperitoneum has been employed in cases involving loss of domain.

The procedure outlined below is the one we are currently following.

The patient is placed in the supine position and covered with a skin drape to prevent direct contact of the prosthetic materials with the skin. The previous scar is removed, and unless it is particularly extensive, a 15 cm incision is sufficient for optimal exposure of the surgical field. Panniculectomy is performed in those cases with very redundant skin and subcutaneous tissue.

Subcutaneous dissection does not extend beyond the hernia defect and the sac is opened as soon as possible longitudinally. The two flaps are preserved until the end of the procedure, determining in advance for each half of the sac which will be left attached to the PRS to help close the posterior layer, or which will be left attached to the anterior rectus sheath to cover the mesh in case of a potential bridge [18,19]. Extensive adhesiolysis is performed throughout the cavity as far as the anterior axillary line and a coloured cloth is placed intraabdominally to protect the intestinal bowels.

The procedure continues with the execution of what we consider an “incomplete Rives” technique. Systematically, the dissection begins with lateral dissection of the retromuscular space, followed by bilateral caudal and then epigastric dissection. Laterally, the entire retromuscular space is dissected until the merge of the neurovascular bundles, which are preserved. This lateral limit has recently been called the “ambivium” [20]. Once we have dissected both lateral retromuscular spaces, then inferiorly, beyond the arcuate line and taking advantage of the distribution of preperitoneal fat [21], dissection continues by dissecting the Retzius space in the midline, the Bogros spaces laterally and exposing Cooper’s ligaments. Attention is given to the epigastric vessels, which, along with the surrounding adipose tissue, are preserved. In the case of an M4 or M5 IH, the spermatic vessels and vas deferens (or round ligament) are parietalised as shown by Stoppa [6].

**FIGURE 1:**
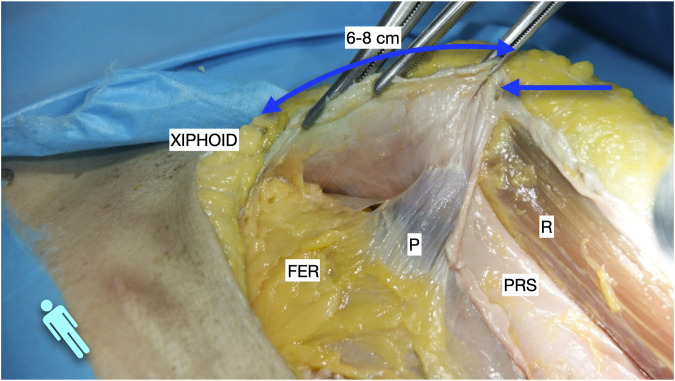
Image of dissection of the preperitoneal pathway in the epigastric area of a defrosted cadaver. An incomplete Rives was performed with preservation of the cranial insertion of the PRS. The blue arrow shows the “incomplete” Rives where the medial incision was stopped at the PRS. The dissection was made leaving the fatty epigastric rhomboid over the peritoneum. The fibres of the TA muscle can be discerned through the fascia transversalis. R, rectus muscle; P, peritoneum; PRS, posterior rectus sheath; FER, fatty epigastric rhomboid; FT, fascia transversalis; A, ambivium; TA, transversus abdominis.

**FIGURE 2:**
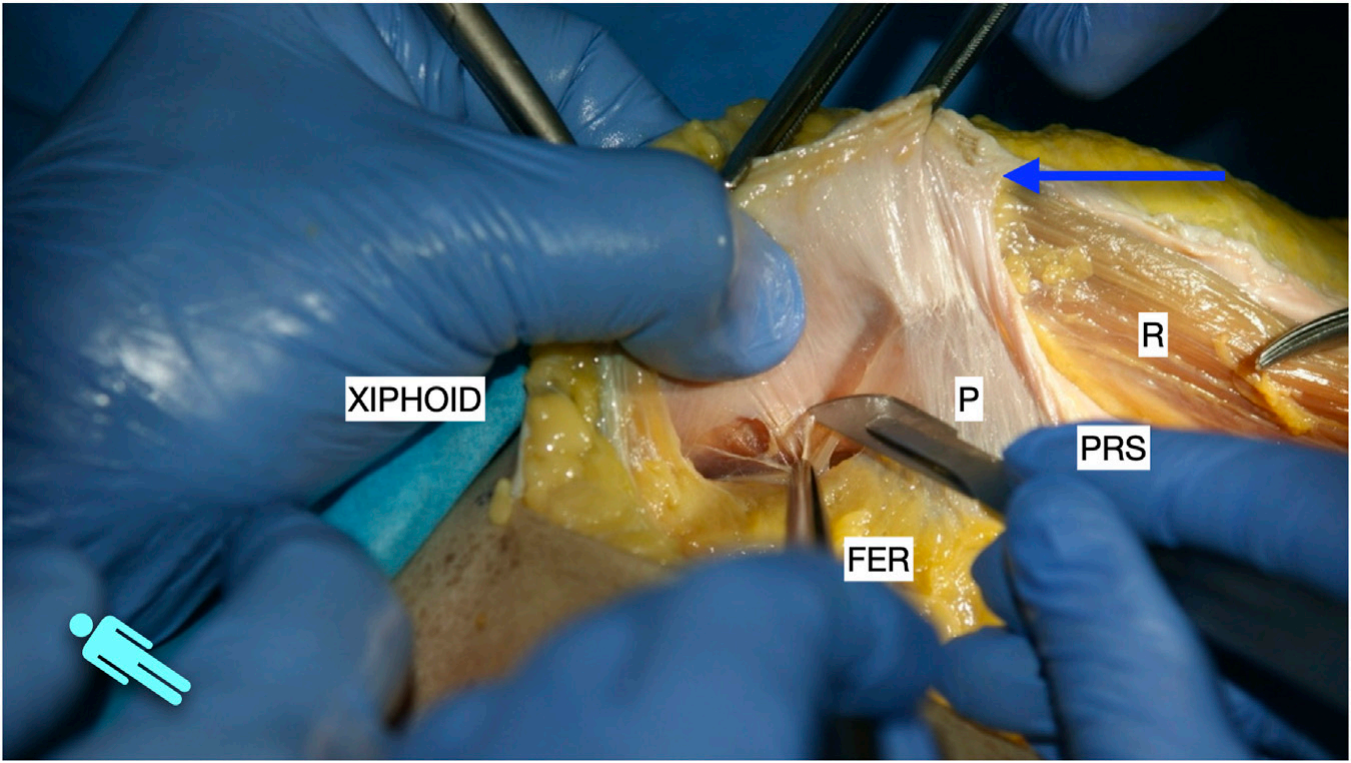
Image of dissection of the preperitoneal pathway in the epigastric area of a defrosted cadaver. Lateral to the FER, the dissection had to be changed to a pre-transversalis plane. The image shows where to start to enter pre-transversalis fascia.

**FIGURE 3:**
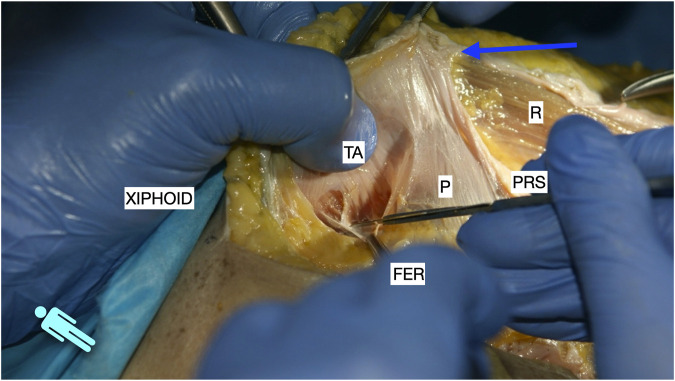
Image of dissection of the preperitoneal pathway in the epigastric area of a defrosted cadaver. The fascia transversalis was left on the floor of the dissection and the TA muscle is shown.

**FIGURE 4:**
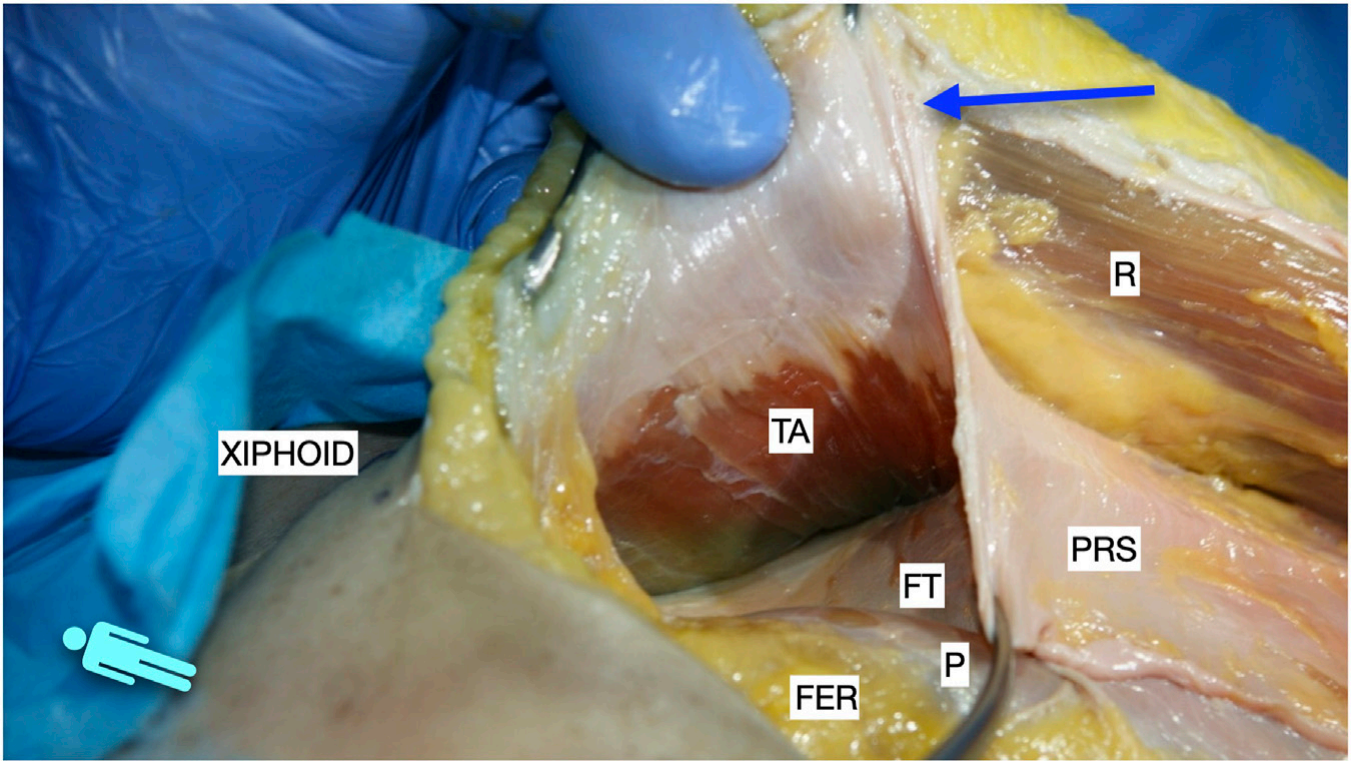
Image of dissection of the preperitoneal pathway in the epigastric area of a defrosted cadaver. The pre-transversalis plane was developed posterior to the PRS, without incising the TA muscle fibres.

Cranially, the medial incision on the PRS stops 7-8 cm from the xiphoid, preserving the anatomical insertion of the PRS on the costal cartilages [Figure 1]. In this epigastric region, the dissection continues laterally and cranially into the preperitoneal space, leaving the lateral fatty tissue of the epigastric rhomboid fat over the peritoneum and navigating just below the “white” PRS. We do both sides first and then, we enter the subxiphoid space. Two centimetres outside the midline, the preperitoneal plane is changed to a pre-transversalis plane under the fibres of the TA muscle [Figures 2–4]. Cranially, the dissection under the fibres of the TA muscle is followed by a pre-facia diaphragmatic plane, under the fibres of the diaphragm. Anatomical findings have shown that, at this level, the pre-transversalis fascia and pre-diaphragmatic fascia planes are preferable to the preperitoneal one. The reason is that here we lose the protection of the preperitoneal fat distribution, with the risk of peritoneal tears. At this phase, the two planes obtained bilaterally converge in the subxiphoid space. Here, a significant adipose pad is systematically left attached to the xiphoid process and the dissection continues beneath it, over the peritoneum. This fatty pad has previously been referred to as the fatty triangle [22]. One constant vessel runs on both sides of this fatty pad, which can be easily controlled. The dissection continues cranially up to the central tendon of the diaphragm. Following the dome shape of the diaphragm is crucial to avoid iatrogenic Morgagni hernias. Particular attention must be paid to the constant anatomical insertion of the fibres of the diaphragm on the PRS. When we reach the central tendon, the fascia diaphragmatica fuses the tendon and our layer becomes again the preperitoneal plane and, therefore, is easy to tear. This entire epigastric preperitoneal dissection entered the plane under the TA muscle in both upper quadrants.

**FIGURE 5:**
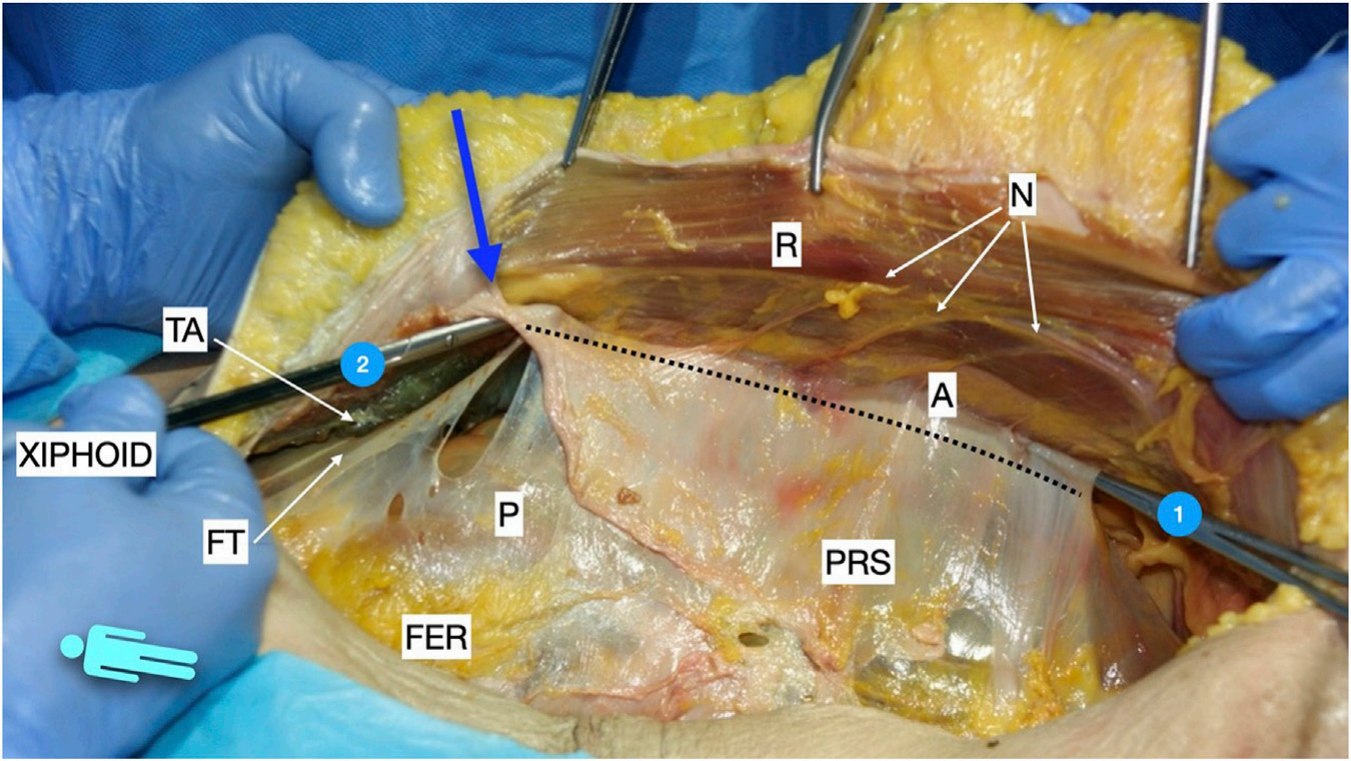
Picture taken in a defrosted cadaver to show the pathways of the preperitoneal space before performing the Madrid PCS. 1: preperitoneal pathway in the Bogros space; 2: preperitoneal pathway in the epigastric area in a pre-transversalis layer. The blue arrow shows the “incomplete Rives” where the medial incision was stopped at the PRS. The dotted line shows the lateral incision at the posterior rectus sheath in the Madrid PCS. FER, fatty epigastric rhomboid; R, rectus muscle; PRS, posterior rectus sheath; P, peritoneum; A, ambivium; N, terminal branches of intercostal nerves; FT, fascia transversalis; TA, transversus abdominis.

**FIGURE 6:**
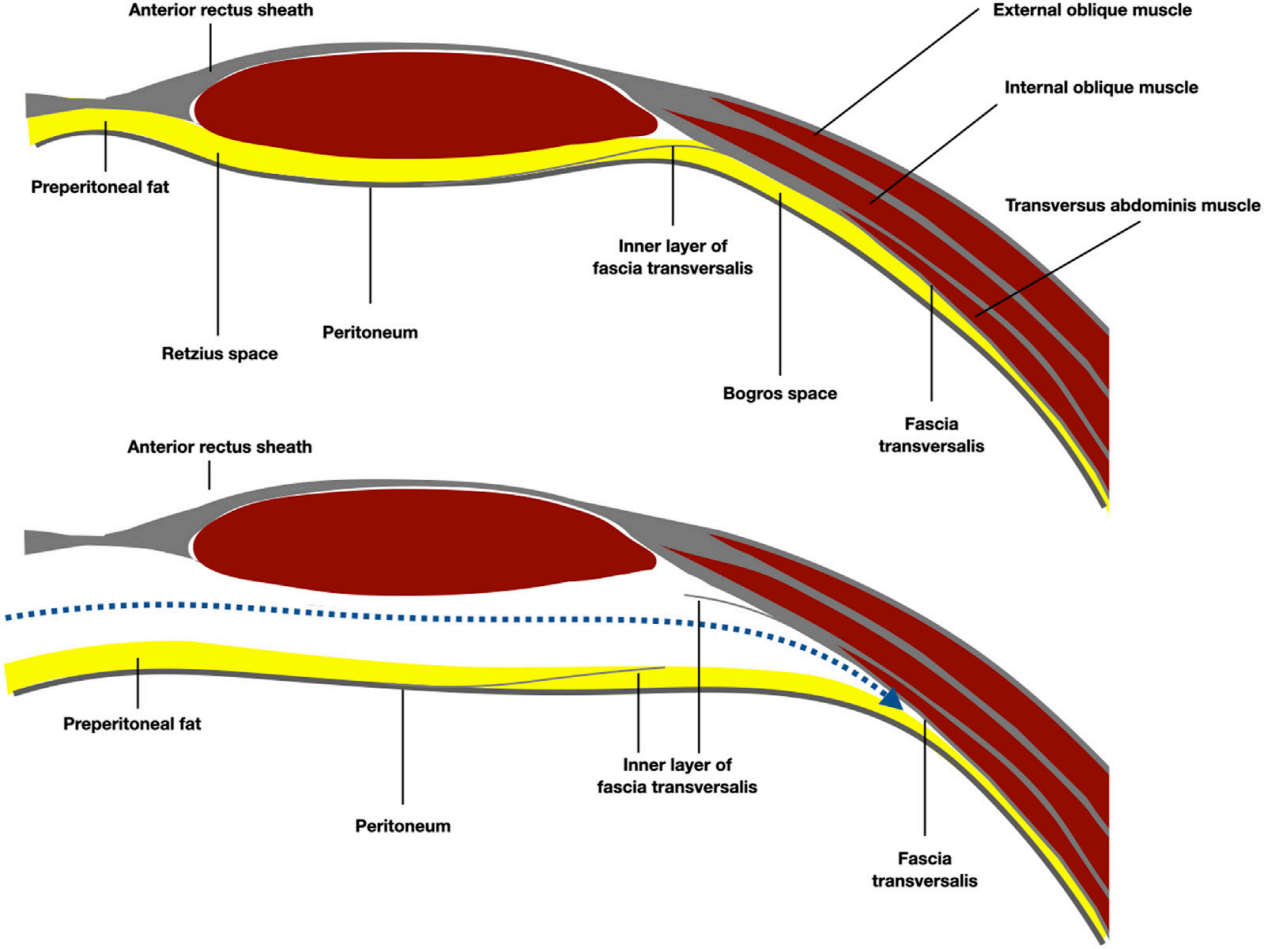
Schematic representation of the anatomy of the Bogros space, under the arcuate line. The preperitoneal plane was developed preperitoneal over the fatty trident.

**FIGURE 7:**
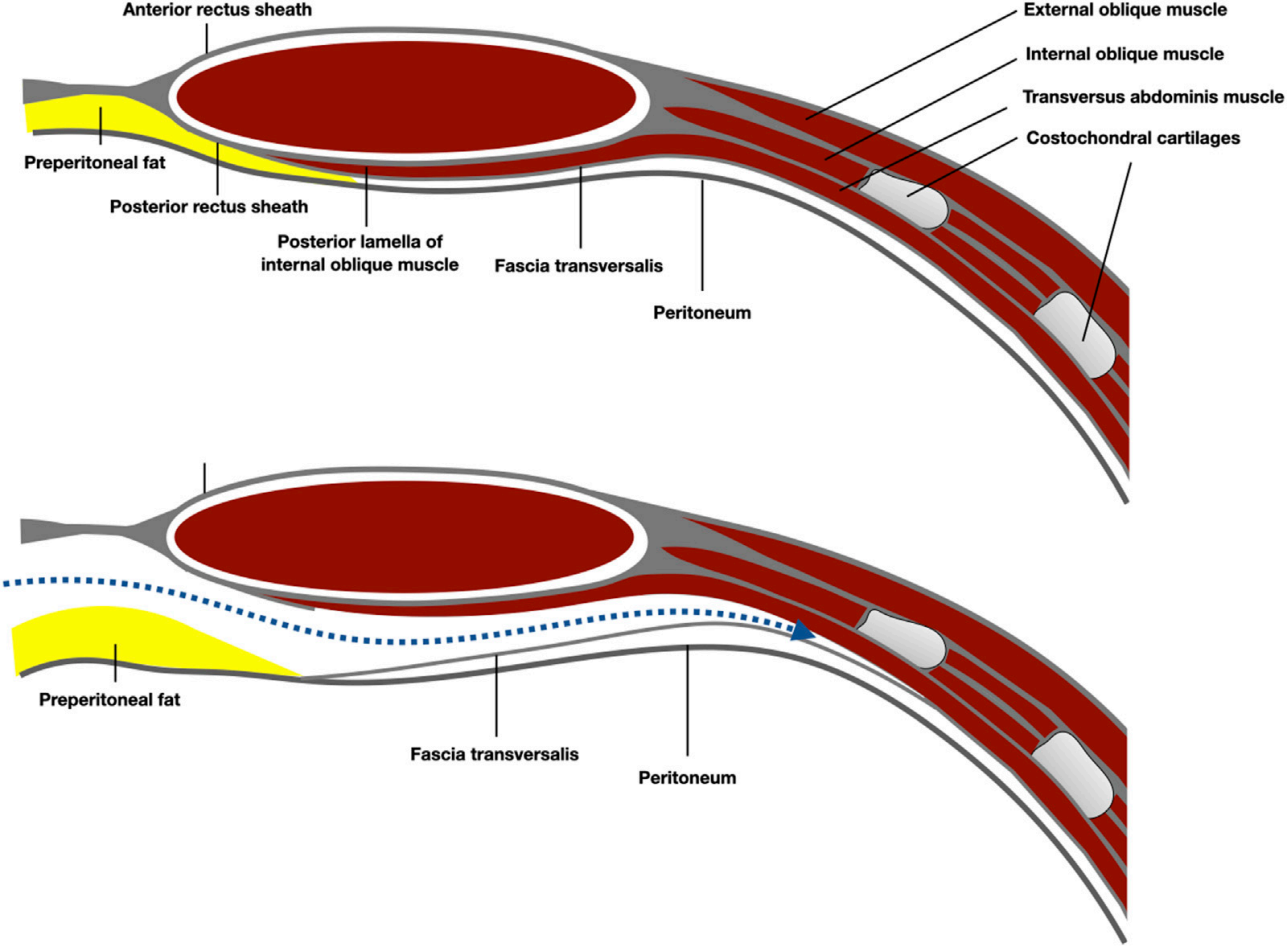
Schematic representation of the anatomy of the epigastric area and the preperitoneal plane developed pre-transversally.

Therefore, the procedure continues with the PRS release. To enter the preperitoneal Bogros space, some fibres of the inner fascia transversalis must be torn or broken. Once in the Bogros space, the arcuate line is identified and with the assistance of a finger, a blunt dissection is performed to access the lateral preperitoneal space. By pushing the visceral sac downward and medially, the peritoneal sac can be separated bluntly from the PRS. A down to up PRS release is performed to join the two dissected preperitoneal pathways: the epigastric pre-transversalis and the Bogros preperitoneal one [Figure 5]. Once the first centimetres are cut 0.5–1 cm medial to the ambivium, we carefully dissect laterally and cranially with gentle manoeuvres on the preperitoneal space under the TA muscle to release the tension on the peritoneum. The down to up PRS release advanced cranially parallel to the ambivium up to the umbilical area, always combining the incision with the previous lateral preperitoneal dissection. Subsequently, the direction becomes oblique to the midline to meet with the point where we stopped the medial incision on the PRS in the epigastric area. After complete PRS release, the preperitoneal dissection continues laterally until the identification of the tip of the twelfth rib cranially, the psoas muscles, and the posterior iliac crest caudally. At this level, it is common to coagulate the constant deep circumflex vessels arising from the iliopsoas muscle. The dissection plane in the lower two-thirds of the abdomen is preperitoneal [Figure 6], while in the upper third, as mentioned earlier, it is pre-transversalis fascia and pre-diaphragmatic fascia [Figure 7]. A horizontal line of fascia transversalis can always be observed between the upper third pre-transversalis and the two lower thirds preperitoneal.

Finally, the abdominal wall reconstruction is carried out. The PRS, along with the peritoneum and the preserved hemi-sac, is used to close the posterior wall in the midline with a continuous slowly-absorbable 00 or 000 monofilament suture. If the posterior wall cannot be closed, a bridge repair using a piece of absorbable mesh is made. All openings larger than 0.5 cm are closed. Subsequently, a 20 × 30 cm bioabsorbable mesh (GORE^®^ BIO-A^®^ Tissue Reinforcement, WL Gore & Associates, Inc. Flagstaff, AZ, United States) is positioned without fixation as a reinforcement of the posterior layer. This mesh is tailored to fit the shape of the inguinal region. Above it, in the same retromuscular-preperitoneal space, an extensive 50 × 50 cm macroporous polypropylene mesh (Bulevb^®^, Dipro Medical Devices SRL, Torino, Italy) is placed and fixed only to Cooper’s ligaments with long-term absorbable sutures. The mesh is placed in a diamond shape for larger patients. In M4-M5 defects, or in the presence of inguinal hernias, this mesh is given the Stoppa configuration to protect the myopectineal areas [6].

Subsequently, anaesthesia of the muscle plane is performed by infiltrating levobupivacaine between the internal oblique and TA muscles. In younger patients or those who are physically active, we usually reinsert the lateral border of the PRS cut to the mesh with running sutures of slowly-absorbable material. If closure of the anterior layer is not possible, the borders of the bridge are sutured with running sutures and covered with a peritoneal flap. In bridges larger than 4 cm in width, we suture an additional sheet of mesh to the bridged area as an inlay. At least one suction drainage is always placed in the retromuscular-periprosthetic space.

### Statistics

The description of variables and statistical analysis were conducted using Microsoft^®^ Excel^®^ for Microsoft 365 MSO (Version 2,312 Build 16.0.17126.20132) 64-bit. Quantitative variables were expressed as mean and range, while categorical variables were presented as absolute frequencies and percentages.

## Results

A total of 223 patients underwent surgery, including 137 men (61.4%) and 86 women (38.6%). The mean age was 63.4 years (range 32–87). A total of 100 patients had a BMI >30 kg/m^2^ with a mean of 33.3 (range 23–40). In total, 84.8% of patients (*n* = 189) had at least one comorbidity, with the most common being arterial hypertension (48.9%), a history of oncological pathology (32.3%), and diabetes (24.2%). A 21.5% of patients were active smokers, while 26.9% had quit smoking less than 12 months before. The mean CeDAR was 36.9%, with 150 patients (67.3%) falling between 30% and 60%. The median ASA score was 2, with the majority of patients being ASA2 and ASA3, 123 (55.2%) and 78 (35%), respectively. Table 1 shows the origin of IH. Of the total enrolled patients, 105 (48.4%) had already undergone abdominal wall repair, with a mean of 2.3 attempts (range 0–13) [Table 1].

**TABLE 1:** Demographics.

Variable	N (%)
Age, years	63.39 (range 32–87)
Sex	137 (61.4%) male; 86 (38.6%) female
BMI, kg/m^2^	33.25 (range 23–40)
ASA median	2
I	17 (7.62%)
II	123 (55.16%)
III	78 (34.98%)
IV	5 (2.24%)
CeDAR mean	36.89 (range 2–91)
<30%	54 (24.22%)
30%–60%	150 (67.27%)
>60%	19 (8.52%)
Comorbidities	
Any	189 (84.75%)
Smocking	48 (21.53%) daily; 60 (26.91%) ex-smocker
Anticoagulation	45 (20.18%)
Diabetes	54 (24.22%)
Immunosuppression	24 (10.76%)
Lung disease	48 (21.53%)
Hypertension	109 (48.88%)
Neoplasia	72 (32.29%)
Previous abdominal wall hernia operation	105 (48.39%)
Number of previous attempts of IH repair, mean	2.33 (range 0–13)
Cause of first surgery	
Hepatobiliopancreatic	19 (8.52%)
Digestive tube	114 (51.12%)
Gynaecologic	23 (10.31%)
Abdominal wall	28 (12.56%)
Urologic	22 (9.87%)
Cardiac	3 (1.35%)
Post-trauma	9 (4.04%)
Vascular	2 (0.89%)
Orthopaedic	1 (0.45%)
Others	2 (0.89%)

Of the 223 patients, 139 (62.3%) had a pure midline defect, while 84 (37.7%) also presented with an associated lateral defect, as illustrated in Table 2. The midline defects were always W3, whereas the cases in which a lateral defect was associated with the midline ones were W1 in 21 cases (24.7%), W2 in 60 cases (71.8%), and W3 in 3 cases (3.6%). The surgical approach included 35 (15.7%) unilateral Madrid PCS and 188 (84.3%) bilateral Madrid PCS procedures. Operative variables are detailed in Table 3, indicating that closure of the posterior layer was consistently achieved, except in 12 patients (5.4%). Bridging of the anterior layer was performed in 76 patients (34.1%), and reinsertion of the transversus abdominis (TA) muscle was conducted in 43 patients (19.3%). The mean operative time was 235.3 min (range 75–540 min).

**TABLE 2:** Characteristics of IH.

Variable	N(%)
Midline defect	139 (62.33%)
Midline + lateral defect	84 (37.67%)
EHS Classification	
M1	3 (1.35%)
M2	7 (3.14%)
M3	14 (6.28%)
M4	2 (0.89%)
M5	1 (0.45%)
M1-2	2 (0.89%)
M1-3	32 (14.35%)
M1-4	19 (8.52%)
M1-5	59 (26.46%)
M2-3	8 (3.59%)
M2-4	29 (13.01%)
M2-5	14 (6.28%)
M3-4	1 (0.45%)
M3-5	28 (12.56%)
M4-5	4 (1.79%)
L1 SUBCOSTAL	21 (9.42%)
L2 FLANK	15 (6.73%)
L3 ILIAC	38 (17.04%)
L4 LUMBAR	10 (4.48%)
Slater Classification	
Minor	12 (5.38%)
Moderate	125 (56.05%)
Major	86 (38.57%)
VHWG Classification	
Grade 1	33 (14.79%)
Grade 2	126 (56.5%)
Grade 3	51 (22.87%)
Grade 4	13 (5.83%)
Wound Classification	
Clean	157 (70.4%)
Clean-Contaminated	44 (19.73%)
Contaminated	13 (5.83%)
Dirty	9 (4.04%)
VHSS Classification	
Stage 1	50 (22.42%)
Stage 2	119 (53.36%)
Stage 3	54 (24.22%)

**TABLE 3:** Operative data.

Variable	N (%)
Elective surgery	222 (99.55%)
Emergency surgery	1 (0.45%)
Size of defect of anterior layer	
Horizontal, cm, mean	12.68 (range 4–30)
Vertical, cm, mean	15.56 (range 5–40)
Surgical technique	
Unilateral Madrid PCS	35 (15.7%)
Bilateral Madrid PCS	188 (84.31%)
Bridging of posterior layer^a^	12 (5.38%)
Bridging of anterior layer^b^	76 (34.08%)
Associated surgery to IH repair	179 (80.27%)
Adhesiolysis	126 (56.5%)
Omentum resection	2 (0.89%)
Intestinal resection	9 (4.04%)
Suture of bowel	13 (5.83%)
Intestinal transit reconstruction	7 (3.14%)
Ileostomy closure	1 (0.45%)
Other abdominal operation	21 (9.42%)
Panniculectomy	47 (21.08%)
None	44 (19.73%)
Reimplant of TA	43 (19.29%)
Drains	
Over the mesh	149 (66.82%)
Subcutaneous and over the mesh	72 (32.29%)
Subcutaneous	1 (0.45%)
None	1 (0.45%)
Mean operative time, min	235 (range 75–540)

^a^ Impossibility to completely close peritoneum and/or posterior rectus sheaths.

^b^ Impossibility to completely close linea alba (borders of anterior rectus sheaths).

A total of 139 patients (62.3%) did not experience any postoperative complications [Table 4]. Of the complications reported in 38 patients (17%), postoperative seroma development was noted in 30 patients (13.5%), requiring procedural intervention. Additionally, 12 patients (5.4%) had a postoperative hematoma, with 3 cases (1.4%) necessitating operative management. Surgical site infections (SSI) occurred in 36 patients (16.1%), with 23 (10.3%) superficial, 7 (3.1%) deep, and 6 (2.7%) organ/space infections. Of these, only 1 patient (0.5%) required removal of the infected mesh. The mean length of hospital stay was 10.9 days (range 1–98 days).

**TABLE 4:** Postoperative complications.

Variable	N (%)	Clavien-Dindo >1
Any complication	84 (37.67%)	
Seroma	38 (17.04%)	
- requiring procedural intervention	30 (13.45%)	30 IIIa
Hematoma	12 (5.38%)	
- requiring procedural intervention	3 (1.35%)	2 IIIa; 1 IIIb
SSI	36 (16.14%)	7 II; 28 IIIa; 1 IIIb
- superficial	23 (10.31%)	7 II; 16 IIIa
- organ/space	6 (2.69%)	6 IIIa
- deep	7 (3.14%)	6 IIIa; 1 IIIb
Wound dehiscence	7 (3.14%)	
Abdominal complications		
Ileus	22 (9.87%)	
Intestinal obstruction	2 (0.89%)	
Fistula	10 (4.48%)	7 IIa; 2 IIIa; 1 IIIb
Intra-abdominal hypertension (IAP) > 11 mmHg	9 (4.04%)	9 IVa
IAP >20 mmHg + organ failure	1 (0.45%)	1 IVa
Systemic complications		
Urinary infection	10 (4.48%)	1 II
Venous line infection	6 (2.69%)	4 II; 2 IIIa
Respiratory failure	16 (7.18%)	5 II; 3 IVa; 4 IVb
Pneumonia	9 (4.04%)	5 II; 1 IIIa; 2 IVb
Cardiac complication	13 (5.83%)	8 II; 3 IVb
Intensive Care Unit stay	89 (39.91%)	
Lenght of hospital stay, day, mean	10.92 (range 1–98)	
Readmission	19 (8.52%)	
Follow-up	199 (89.24%)	
Lost to follow-up	10 (4.48%)	
Deceased due to unrelated causes	14 (6.28%)	
Duration of follow-up, day, mean	718 (range 180–2,216)	
Late SSI		
- superficial	0	
- deep wound infection	2 (0.94%)	2 IIIa
- mesh infection	5 (2.35%)	5 IIIb
Chronic seroma	6 (2.69%)	5 IIIa; 1 IIIb
Chronic pain		
- occasionally need for pain treatment	7 (3.29%)	
- daily pain treatment	2 (0.94%)	
- discomfort	6 (2.82%)	
Bulging		
- symptomatic	2 (0.94%)	
- asymptomatic	14 (6.57%)	
Foreign body reaction	2 (0.89%)	
Recurrence	4 (1.88%)	2 IIIb

A total of 199 patients (89.24%) completed at least a 6-month clinical follow-up [Table 4]. In 4 cases (1.8%), clinical follow-up was not feasible, necessitating a telephone interview. The mean follow-up duration was 718 days (range 180–2,216 days). During follow-up, 14 patients (6.3%) died due to causes unrelated to surgery, while an additional 10 patients (4.5%) did not attend regular check-ups. Late complications included 7 patients (3.3%) experiencing deep wound or prosthesis infections, requiring surgery in 5 cases (2.4%). Chronic seroma developed in 6 patients (2.7%), and a foreign body reaction was observed in only 2 patients (0.9%). Chronic pain was reported by 15 patients (7%), with 2 subjects (0.9%) requiring daily pain treatment. Patients with uncertain clinical signs of recurrence underwent a follow-up CT scan, which revealed a total of 4 recurrences (1.9%).

## Discussion

The PCS with TAR is a technique described to repair large midline hernias where the Rives-Stoppa technique is insufficient for abdominal wall reconstruction. This technique, as outlined by Novitsky et al, allows for the successful treatment of large IH, requiring extensive dissection, while maintaining the advantage of using a permanent prosthesis in the sublay position [7]. The results reported in their case series are very favourable, despite the fact that approximately 90% of the patients had a grade 2-3 IH based on the modified hernia grading scale and a median hernia width of 15 cm. In a subsequent study, this group reported a low number of recurrences (3.7%) with a complete closure rate of the anterior layer of the abdominal wall of 97% [23]. Zolin et al. reported a 92% success rate in closing the anterior layer, with a composite hernia recurrence rate of 26% in a case series of 1,203 patients, 57% of whom had recurrences and a median hernia width of 15 cm [24]. The effectiveness of this technique in terms of recurrence was also reported by Winder et al. In their study, although with a smaller group of patients, the authors reported a 2.7% recurrence rate [25]. Heniford et al. confirmed these results in their study of 1,023 patients in whom PCS with TAR was performed in case of dissection difficulties with the pure preperitoneal technique, reporting a 5% recurrence rate [10]. Finally, Sagnelli et al, in their recent study, reported excellent results regarding the effectiveness of the technique. In their case series of 117 patients with complex IH, PCS with TAR was performed, and the abdominal wall was reconstructed with a double prosthesis, following the Madrid APPROACH, with a reported recurrence rate of less than 1% [13]. In this current case series, where the Madrid PCS was performed, we report a recurrence rate of 1.9%, lower than most studies in the literature, confirming that this technique is a valid alternative to the original one.

**FIGURE 8:**
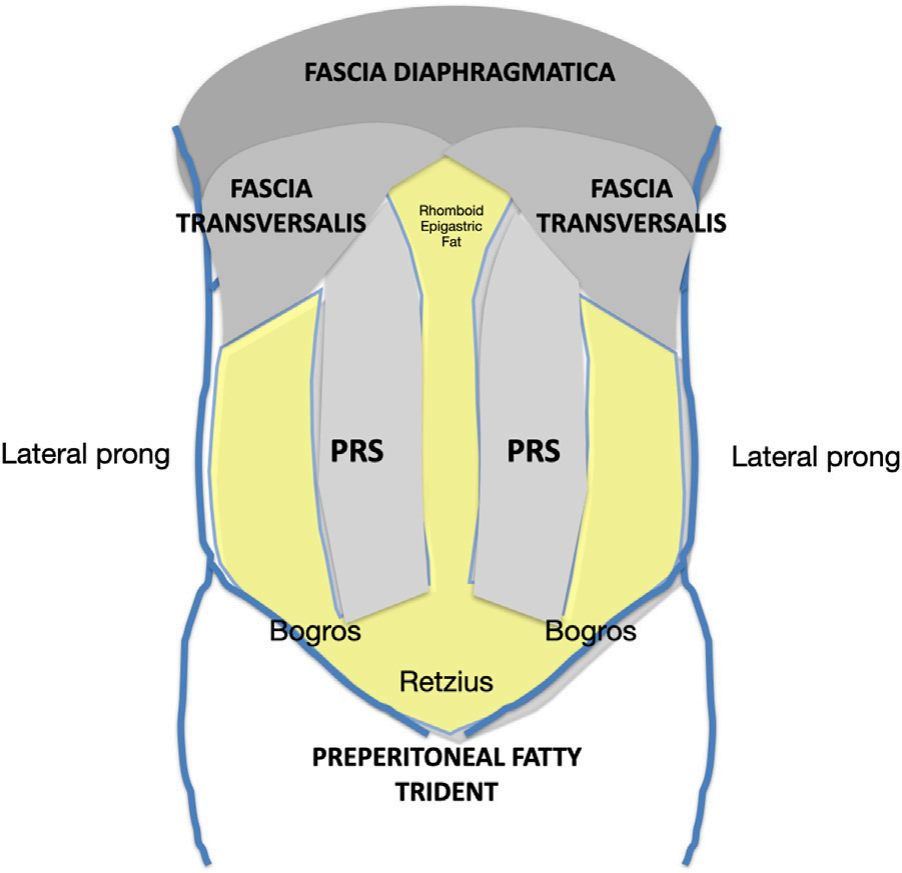
Schematic of the retromuscular and preperitoneal space dissected according to the Madrid PCS. PRS, Posterior rectus sheath.

One of the key points of the Madrid PCS is the reconstruction using very large meshes, applying Stoppa´s concept of “giant reinforcement of the visceral sac” to the midline IH [6]. The space for this mesh is obtained by a wide dissection over the parietal peritoneum and under the overlying abdominal wall muscles: from Cooper’s ligament to the central tendon, and from the tip of the twelfth rib and the psoas muscles to the contralateral ones. Anatomical findings have shown that this vast retromuscular and preperitoneal space includes the PRS, the preperitoneal trident, the parietal peritoneum, the fascia transversalis, and the fascia diaphragmatica[Figure 8]. This thin layer is referred to as“the posterior layer” in PCS techniques. Its use provides sufficient extension and overlap to effectively repair large defects in the midline and the combination of midline and lateral ones [26]. The difference with complete preperitoneal dissection [10,27] is that with the Madrid PCS, the medial and lateral release facilitates the midline closure of large defects.

**FIGURE 9:**
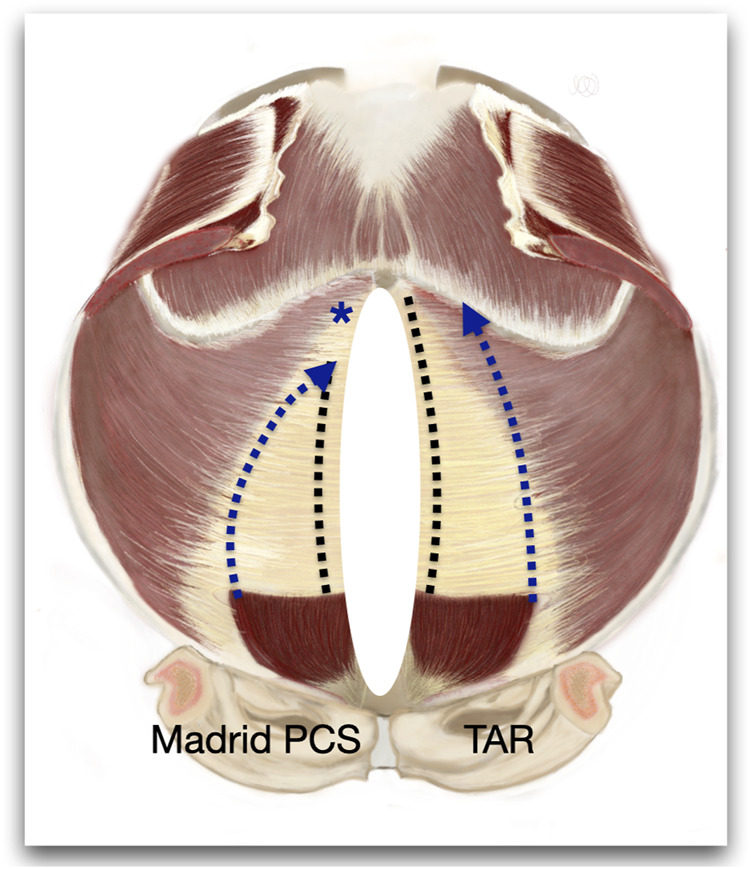
Figure of an internal view of the abdominal wall to represent the differences between the Madrid PCS and TAR. * Shows the preservation of the cranial insertion of the PRS.

**FIGURE 10:**
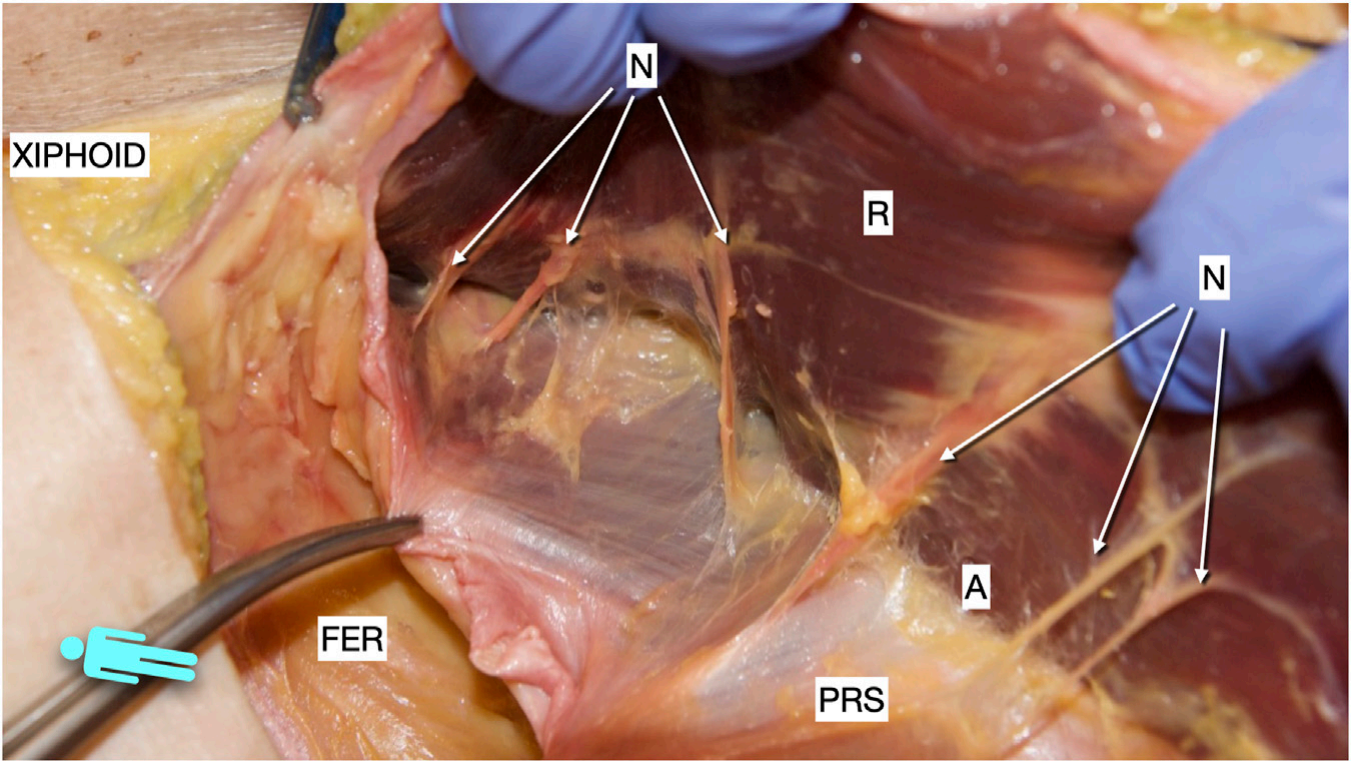
Picture taken of a defrosted cadaver in which an incomplete Rives was dissected. It shows the medial arise of the medial merge of the nerves in the epigastric area. R, rectus muscle; PRS, posterior rectus sheath; FER, fatty epigastric rhomboid; A, ambivium; N, terminal branches of intercostal nerves.

Although the Madrid PCS was initially considered a modification of the TAR [11, 12, 14, 28], the significant anatomical differences probably suggest that it should be categorised as a PRS release rather than a TAR. These differences are: first, the preservation of the PRS insertion, and second, the lateral release of the PRS without cutting the TA muscle [Figure 9]. The first aspect involves the cranial preservation of the PRS at its physiological attachment to the chondrocostal cartilage. Anatomical dissections in the cadaver laboratory and experience in performing PCS have shown us that there is a close anatomical and, therefore, functional relationship between the PRS, the diaphragm, and the TA muscle. We have observed that the fibres of the diaphragm invariably insert at the PRS. Therefore, maintaining the integrity of the PRS avoids injury to these diaphragmatic fibres. Consequently, it seems convenient to change to a preperitoneal plane in the subxiphoid area. When we started learning the TAR technique, we became aware that the terminal branches of the T7, T8, and T9 intercostal nerves arise more medially than previously reported and they are difficult to preserve unless the TAR is performed very medially [Figure 10]. It is not so uncommon to see muscle atrophy of the rectus muscle in CT controls and patients complaining of a bulge. We then decided to perform the lateral release of the PRS in the upper third, following the myofascial limit of the TA muscle. Since this medial release is more difficult to perform than a TAR, we have standardised the technique with our recommendation to follow the pathways of the preperitoneal plane before starting the lateral release. Therefore, before any release is made, we recommend entering the preperitoneal plane starting at the Bogros space and pre-transversalis fascia in the subxiphoid area. As explained previously [21], the preperitoneal fat distribution allows entering the plane under the TA muscle without any lateral release at the PRS. Finally, the Madrid PCS is a technique halfway between Novitsky´s TAR and Heniford´s preperitoneal repair [7,9,10]. Furthermore, preserving the TA muscle cranially may contribute to the stabilisation and mobilisation of the trunk.

Another point of discussion is the difference in midline approximation obtained comparing the Madrid PCS and TAR. We certainly think that, from an anatomical point of view, we are also performing a release at the insertion of the TA muscle, and the difference must only be in the cranial preservation of the PRS. Anatomical studies in the cadaver laboratory may reveal if there is a substantial difference, although we agree that these cadaver studies may have significant limitations [29].

Despite good results in terms of recurrence rate, any complex abdominal wall repair is not free from complications. Novitsky et al, in their study, reported an SSE rate of 18% and an SSI rate of 9% with a mean length of hospital stay of 6 days [23]. Heniford et al. reported a 27% SSE rate and a 15% SSI rate with a mean length of hospital stay of 5 days [10]. However, in a subsequent study, the same authors demonstrated how experience can significantly improve the complication rate (from 26% to 13%) and the recurrence rate (from 7% to 2%) [27]. Sagnelli et al. also showed complications that were in line with those reported in the Literature. They reported a seroma rate of 26%, a hematoma rate of 17%, and only a 3.4% SSI rate [13]. Slightly better results were obtained by Zolin et al, who reported only 8% of SSOPI in patients with more than 1 year of follow-up [24]. Finally, a recent meta-analysis, including 5 studies from 2016 to 2017, reported similar results, with an average SSO rate of 15% and an SSI rate of 7% [30].

In our case series, 38% of patients experienced a complication, either systemic or related to the surgical site. The reported rates of seroma and SSI were 17% and 16%, respectively. Of these, only one patient, who also underwent simultaneous intestinal transit reconstruction, required reoperation to completely remove the infected mesh. With regard to late infections, 7 (3%) patients were readmitted for treatment. Of these, 5 patients had a mesh infection, and in all cases, the prosthesis was removed. Compared to other studies, our results regarding postoperative complications were higher, which could be influenced by the patient´s non-ideal preoperative conditions and the careful collection of prospective data. Approximately 85% of our patients had at least one comorbidity, and 67% had a CeDAR score of developing a surgical site complication between 30% and 60%. Furthermore, 53% and 24% of the patients were classified as grade 2 and 3 according to the Ventral Hernia Staging System (VHSS) classification [31].

There are several notable limitations. There is no comparison group. This design choice limits the ability to assess the relative effectiveness and safety of the Madrid PCS compared to alternative surgical approaches. However, we do consider this approach to be the most anatomically respectful. The population treated at three specialised centres also limits its applicability to other centres not dedicated to the abdominal wall. The study may be subject to selection bias since patients were recruited from specialised centres, and those with more complex cases or comorbidities may be overrepresented. This could impact the external validity of the findings. On the other hand, the selection criteria for enrolling patients avoid selection bias and the application of the same protocol prevents the bias of misclassification. Finally, we recognise an inherent publication bias due to the tendency to publish positive results, potentially leading to an overestimation of the effectiveness of the Madrid PCS. technique. Nonetheless, this study offers a large sample reporting favourable long-term outcomes demonstrating the durability and sustained effectiveness of the Madrid PCS in addressing midline incisional hernias. Additionally, the main aim of this study was to provide a comprehensive description of the surgical technique based on anatomical findings. This knowledge is considered crucial for surgeons, suggesting that a thorough anatomical approach contributes to the success of this technique.

## Conclusion

The Madrid PCS stands out as a technique that facilitates the reconstruction of large IHs with remarkable efficacy in preventing recurrence. This approach introduces a technical variation rooted in the anatomical study of the abdominal wall and the arrangement of its preperitoneal fat. These modifications not only improve the intuitive execution of the technique but also foster a more respectful approach to the musculofascial and nervous components of the anterior abdominal wall. Furthermore, the Madrid PCS allows for the placement of large prostheses in the retromuscular-preperitoneal space, aligning with the fundamental principle of the giant prosthetic reinforcement of the visceral sac. This adherence contributes to a low incidence of long-term recurrences, contributing to the favourable outcomes associated with the technique.

## Data Availability

The datasets presented in this article are not readily available because the authors confirm that the data supporting the findings of this study are available within the article (and/or) its supplementary materials. Requests to access the datasets should be directed to cellodeluca@gmail.com.

## References

[1] Deerenberg EB Harlaar JJ Steyerberg EW Lont HE van Doorn HC Heisterkamp J Small Bites Versus Large Bites for Closure of Abdominal Midline Incisions (STITCH): A Double-Blind, Multicentre, Randomised Controlled Trial. Lancet (2015) 386(10000):1254–60. 10.1016/s0140-6736(15)60459-7 26188742

[2] Cobb WS Warren JA Ewing JA Burnikel A Merchant M Carbonell AM. Open Retromuscular Mesh Repair of Complex Incisional Hernia: Predictors of Wound Events and Recurrence. J Am Coll Surg (2015) 220(4):606–13. 10.1016/j.jamcollsurg.2014.12.055 25797746

[3] Muysoms FE Antoniou SA Bury K Campanelli G Conze J Cuccurullo D European Hernia Society Guidelines on the Closure of Abdominal Wall Incisions. Hernia (2015) 19:1–24. 10.1007/s10029-014-1342-5 25618025

[4] Slater NJ Montgomery A Berrevoet F Carbonell AM Chang A Franklin M Criteria for Definition of a Complex Abdominal Wall Hernia. Hernia (2014) 18(1):7–17. 10.1007/s10029-013-1168-6 24150721

[5] Rives J Pire JC Flament JB Polot JP. Major Incisional Hernias. In: Chevrel JP, editor. Surgery of the Abdominal wall. New York: Springer-Verlag (1987). p. 116–44.

[6] Stoppa RE. The Treatment of Complicated Groin and Incisional Hernias. World JSurg (1989) 13(5):545–54. 10.1007/BF01658869 2683400

[7] Novitsky YW Elliott HL Orenstein SB Rosen MJ. Transversus Abdominis Muscle Release: A Novel Approach to Posterior Component Separation During Complex Abdominal Wall Reconstruction. Am J Surg (2012) 204(5):709–16. 10.1016/j.amjsurg.2012.02.008 22607741

[8] Carbonell AM Cobb WS Chen SM. Posterior Components Separation During Retromuscular Hernia Repair. Hernia (2008) 12(4):359–62. 10.1007/s10029-008-0356-2 18293053

[9] Novitsky YW Porter JR Rucho ZC Getz SB Pratt BL Kercher KW Open Preperitoneal Retrofascial Mesh Repair for Multiply Recurrent Ventral Incisional Hernias. J Am Coll Surg (2006) 203(3):283–9. Epub 2006 Jul 13. PMID: 16931299. 10.1016/j.jamcollsurg.2006.05.297 16931299

[10] Heniford BT Ross SW Wormer BA Walters AL Lincourt AE Colavita PD Preperitoneal Ventral Hernia Repair: A Decade Long Prospective Observational Study With Analysis of 1023 Patient Outcomes. Ann Surg (2020) 271(2):364–74. 10.1097/SLA.0000000000002966 30080725

[11] Robin A Blazquez Hernando L Lopez-Monclus J Cruz Cidoncha A San Miguel Méndez C Jimenez Cubedo E How We Do it: Down to up Posterior Components Separation. Langenbecks Arch Surg (2018) 403(4):539–46. Epub 2018 Mar 3. PMID: 29502282. 10.1007/s00423-018-1655-4 29502282

[12] García-Ureña MÁ López-Monclús J Cuccurullo D Blázquez Hernando LA García-Pastor P Reggio S Abdominal Wall Reconstruction Utilizing the Combination of Absorbable and Permanent Mesh in a Retromuscular Position: A Multicenter Prospective Study. World J Surg (2019) 43(1):149–58. PMID: 30132226. 10.1007/s00268-018-4765-9 30132226

[13] Sagnelli C Tartaglia E Guerriero L Montanaro ML D'Alterio G Cuccurullo D Long-Term Outcomes of Madrid Approach After TAR for Complex Abdominal Wall Hernias: A Single-Center Cohort Study. Hernia (2023) 25:1355–61. Epub ahead of print. 10.1007/s10029-020-02273-9 37726424

[14] Reinpold W. Transversus Abdominis Muscle Release: Technique, Indication, and Results. Int J Abdom Wall Hernia Surg (2018) 1(3):79–86. 10.4103/ijawhs.ijawhs_27_18

[15] Muysoms FE Miserez M Berrevoet F Campanelli G Champault GG Chelala E Classification of Primary and Incisional Abdominal Wall Hernias. Hernia (2009) 13(4):407–14. 10.1007/s10029-009-0518-x 19495920 PMC2719726

[16] Dindo D Demartines N Clavien PA. Classification of Surgical Complications: A New Proposal With Evaluation in a Cohort of 6336 Patients and Results of a Survey. Ann Surg (2004) 240(2):205–13. 10.1097/01.sla.0000133083.54934.ae 15273542 PMC1360123

[17] von Elm E Altman DG Egger M Pocock SJ Gotzsche PC Vandenbroucke JP The Strengthening the Reporting of Observational Studies in Epidemiology (STROBE) Statement: Guidelines for Reporting Observational Studies. Int J Surg (2014) 12:1495–9. 10.1016/j.ijsu.2014.07.013 25046131

[18] Malik A Macdonald AD de Beaux AC Tulloh BR. The Peritoneal Flap Hernioplasty for Repair of Large Ventral and Incisional Hernias. Hernia (2014) 18(1):39–45. Epub 2013 Apr 9. PMID: 23568492. 10.1007/s10029-013-1086-7 23568492

[19] Nielsen MF de Beaux A Tulloh B. Peritoneal Flap Hernioplasty for Reconstruction of Large Ventral Hernias: Long-Term Outcome in 251 Patients. World J Surg (2019) 43(9):2157–63. PMID: 31065774. 10.1007/s00268-019-05011-0 31065774

[20] Vierstraete M Pereira Rodriguez JA Renard Y Muysoms F. EIT Ambivium, Linea Semilunaris, and Fulcrum Abdominalis. J Abdom Wall Surg (2023) 2:12217. PMID: 38312427; PMCID: PMC10831682. 10.3389/jaws.2023.12217 38312427 PMC10831682

[21] Garcia-Urena MÁ Lopez-Monclus J de Robin Valle de Lersundi A Blazquez Hernando LA Medina Pedrique M Rial Justo X Pathways of the Preperitoneal Plane: From the "Fatty Triangle" in Rives to the "Fatty Trident" in Extended Retromuscular Abdominal Wall Reconstruction. A Tribute to Prof. Schumpelick. Hernia (2023) 27(2):395–407. 10.1007/s10029-022-02602-0 35426573

[22] Conze J Prescher A Klinge U Saklak M Schumpelick V. Pitfalls in Retromuscular Mesh Repair for Incisional Hernia: The Importance of the "Fatty Triangle. Hernia (2004) 8(3):255–9. Epub 2004 Jun 5. PMID: 15185126. 10.1007/s10029-004-0235-4 15185126

[23] Novitsky YW Fayezizadeh M Majumder A Neupane R Elliott HL Orenstein SB. Outcomes of Posterior Component Separation With Transversus Abdominis Muscle Release and Synthetic Mesh Sublay Reinforcement. Ann Surg (2016) 264:226–32. PMID: 26910200. 10.1097/SLA.0000000000001673 26910200

[24] Zolin SJ Krpata DM Petro CC Prabhu AS Rosenblatt S Rosen S Long-Term Clinical and Patient-Reported Outcomes After Transversus Abdominis Release With Permanent Synthetic Mesh: A Single Center Analysis of 1203 Patients. Ann Surg (2023) 277(4):e900–e906. 10.1097/SLA.0000000000005443 35793810

[25] Winder JS Behar BJ Juza RM Potochny J Pauli EM. Transversus Abdominis Release for Abdominal Wall Reconstruction: Early Experience With a Novel Technique. J Am Coll Surgeons (2016) 223(2):271–8. 10.1016/j.jamcollsurg.2016.04.012 27107825

[26] Munoz-Rodriguez JM Lopez-Monclus J San Miguel Mendez C Perez-Flecha Gonzalez M Robin-Valle de Lersundi A Blázquez Hernando LA Outcomes of Abdominal Wall Reconstruction in Patients With the Combination of Complex Midline and Lateral Incisional Hernias. Surgery (2020) 168(3):532–42. Epub 2020 May 12. PMID: 32527646. 10.1016/j.surg.2020.04.045 32527646

[27] Katzen MM Kercher KW Sacco JM Ku D Scarola GT Davis BR Open Preperitoneal Ventral Hernia Repair: Prospective Observational Study of Quality Improvement Outcomes Over 18 Years and 1,842 Patients. Surgery (2023) 173(3):739–47. 10.1016/j.surg.2022.07.042 36280505

[28] Urena MÁG López-Monclús J Hernando LAB Munoz-Rodriguez J García de León LR Avilés Oliveros A Narbenhernien: Offene Operationsverfahren und Ergebnisse Einer Kohortenstudie mit 343 Patienten [Incisional Hernia: Open Abdominal Wall Reconstruction. Current State of the Technique and Results]. Chirurgie (Heidelb) (2024) 95(1):10–9. 10.1007/s00104-023-02005-6 38157070

[29] Köhler G. Letter to the Editor: Anterior and Posterior Component Separation; Limitations of Cadaver Studies. Am J Surg (2020) 219(4):728–9. Epub 2019 May 25. PMID: 31153583. 10.1016/j.amjsurg.2019.05.010 31153583

[30] Wegdam JA Thoolen JMM Nienhuijs SW de Bouvy N de Vries Reilingh TS. Systematic Review of Transversus Abdominis Release in Complex Abdominal Wall Reconstruction. Hernia (2019) 23(1):5–15. 10.1007/s10029-018-1870-5 30539311

[31] Petro CC O'Rourke CP Posielski NM Criss CN Raigani S Prabhu AS Designing a Ventral Hernia Staging System. Hernia (2016) 20(1):111–7. 10.1007/s10029-015-1418-x 26342924

